# Hydroponic Cultured Ginseng Leaves Zinc Oxides Nanocomposite Stabilized with CMC Polymer for Degradation of Hazardous Dyes in Wastewater Treatment

**DOI:** 10.3390/ma14216557

**Published:** 2021-11-01

**Authors:** Yinping Jin, Ling Li, Reshmi Akter, Esrat Jahan Rupa, Deok-Chun Yang, Se Chan Kang, Hao Zhang

**Affiliations:** 1 Institute of Special Wild Economic Animals and Plants, Chinese Academy of Agricultural Sciences, Changchun 130112, China; jinyinping@caas.cn; 2 Department of Oriental Medicinal Biotechnology, College of Life Science, Kyung Hee University, Yongin-si 17104, Gyeonggi-do, Korea; aqling@naver.com (L.L.); reshmiakterbph57@gmail.com (R.A.); eshratrupa91@gmail.com (E.J.R.); dcyang@khu.ac.kr (D.-C.Y.)

**Keywords:** hydroponic ginseng, o-carboxymethyl chitosan (CMC), ZnO, dye degradation, water treatment

## Abstract

This study demonstrated the synthesis of o-carboxymethyl chitosan (CMC)-stabilized zinc oxide nanocomposites (ZnO NCs) combined with aqueous leaves extracts of hydroponically cultured ginseng and used as a photocatalyst for the degradation of hazardous dyes, including malachite green (MG), rhodamine B (RB), and congo red (CR) under ultraviolet illumination. Hydroponic ginseng leaves contain bioactive components, namely ginsenoside and natural polyphenol, which prompt ginseng’s biological effect. Besides, the CMC polymer is naturally biodegradable, stabilizes the nanoformation and enhances the solubility of ginsenoside. The hydroponic ginseng leaves zinc oxide CMC nanocomposites (GL–CMC–ZnO NCs) were synthesized using the co-precipitation method and characterized using different analytical methods. The FTIR analysis identified significant phytochemicals in the leaves extracts and cotton-shape morphology observed using FE-TEM analysis. The XRD analysis also determined that the crystallite size was 28 nm. The photocatalyst degraded CR, RB, and MG dyes by approximately 87%, 94%, and 96% within contact times of 10, 20, 25, and 30 min, respectively, when the dye concentration was 15 mg/L. As far as our knowledge, this is the first report on hydroponic ginseng NCs incorporated with the CMC polymer for the degradation of hazardous dyes on wastewater treatment. This study can add significant value to large-scale wastewater treatment.

## 1. Introduction

Dyes are widely used in various chemical and textile industries, even though they are known as pollutants. Depending on the mode of disposal, discharge dyes can be hazardous to the surrounding environment and toxic to human beings [1,2]. Extensive use of organic dyes causes the contamination of water and groundwater, increasing risk to aquatic organisms [3]. Malachite green (MB), methyl red, methylene blue, congo red (CR), eosin Y, bromophenol blue, phenol red, methyl orange, and rhodamine B (RB) are the most commonly used industrial organic dyes [4]. Due to the higher stability against light and oxidation reactions, the degradation of industrial dyes is very challenging [5,6]. 

There are numerous treatment methods for removing dyes from wastewater, and approaches including physical, chemical, and biological water treatment have been introduced to remove dyes from wastewater. However, all of them have some disadvantages [7]. The photocatalytic reaction has become a methodical process for mineralizing toxic organic constituents since 1972, because of a potent oxidizing agent such as hydroxyl radical (OH•) [8]. Besides, nanoparticles (NPs) can efficiently absorb dyes owing to high surface-to-volume ratios [9]. In comparison with other NPs, zinc oxide NPs have gained scientific spotlight due to their high photocatalytic efficiency and cost-effective, non-toxicity, effectual, and eco-friendly properties; zinc oxide NPs are known as one of the most essential widely used semiconductor-based photocatalysts. Moreover, because of the high quantum efficiency, ZnO NPs have shown more decomposition of organic pollutants as compared to TiO_2_ NPs [10]. ZnO NPs have some unique characteristics that make ZnO NPs a multifunctional agent and a more prominent option for wastewater treatment. Metal oxide NPs such as TiO_2_, ZnO, SnO_2_, CuO, and Cu_2_O have shown good photocatalytic activity to remove organic pollutants under UV light illumination [2].

Green synthesis of ZnO NPs offers a cost-effective and eco-friendly alternative for eliminating dyes that contribute to environmental problems [11].

ZnO NPs can be prepared following different methods including the sol-gel method [12], co-precipitation method [13], sol-gel spin coating technique [14], and pulsed-laser deposition (PLD) [15]. This study focused on a quicker synthesis method of ZnO NPs (co-precipitation method) using plant phytochemicals and stabilized the NPs with a biodegradable polymer o-carboxymethyl chitosan (CMC). The green synthesis of NPs is considered eco-friendly rather than the chemical synthesis process using plant phytochemicals. In short, most chemical synthesis methods contain toxic chemicals that can lead to dangerous effects on the aquatic system, besides chemical and physical synthesis methods which operate in high temperatures and sometimes inert conditions and are cost-effective. In summary, chemicals and stabilizers lead to serious toxicity after the catalytic reaction that is more dangerous initial one [1]. Here, ginseng leaves ZnO NPs with CMC polymer nanocomposites (NCs) was designed due to uses of ginseng leaves, instead of hazardous toxic chemicals of other conventional methods. 

Previous studies of green synthesized ZnO NPs using *Cordyceps militaris* fungus [16] and *Rubus coreanus* [17] have shown promising photocatalytic activity on dye degradation in wastewater remediation.

*Panax ginseng* (Korean ginseng) is a plant under the family Araliaceae distributed in 35 countries as an herbal medicine for over 2000 years [18]. Ginseng contains more than 100 bioactive components known as ginsenoside [19], which has already been proven to have anti-inflammatory [20], antioxidant [21], and hepatoprotective [22] activities, but there is no evidence for wastewater treatment. This study highlighted hydroponically cultured ginseng, and it has been reported that hydroponically cultured ginseng leaves contain a significantly higher amount of ginsenosides than root and stem [23].

Here, 120-day-old hydroponically cultured fresh ginseng leaves were used to prepare zinc oxide NPs that were stabilized with CMC to enhance the solubility rate of ginsenosides [24], which may further accelerate the degradation rate of toxic dyes. The polymer CMC is a chitosan derivative that can be dissolved in water at high pH and widely used in desalination [25], CO_2_ capture [26], and wastewater treatment [27]. Besides, natural chitosan is not soluble in PH higher than 6.5 [27]. Polymer-based photocatalysts contain NPs and polymers, making NCs more stable, chemically consistent, and have higher mechanical durability [28]. Chitosan-mediated NCs have gained enormous attention due to their biocompatibility, biodegradability, and non-toxic nature [29]. It provides a synergistic effect in ZnO NCs to remove toxic dyes more efficiently from wastewater [30]. The photocatalytic activity of GL–CMC–ZnO NCs was investigated under UV light, where the NCs show excellent photocatalytic activity depredating toxic CR, RB, and MG dyes. The presence of a higher amount of polyphenol and ginsenosides into the fresh leaves of ginseng enhances the photocatalytic activity of GL–CMC–ZnO NCs. Besides, CMC is a natural polymer that increases the solubility rate of ginsenosides that may promote catalytic activity. Due to synergistic effects of both polymers and NPs can serve NCs as a safe, low-cost, and biodegradable material for degrading hazardous dyes from wastewater.

## 2. Materials and Methods

### 2.1. Plant and Chemical

The leaves of hydroponically cultured ginseng were collected from Hanbang Bio Laboratory, Kyung Hee University, South Korea. Zinc nitrate (>98%) and NaOH (>98%) were bought from Dae-Jung Chemicals and Metals Co., Ltd. (Pyeontaek, Korea). All dyes and other reagents were supplied by Sigma-Aldrich, St. Louis, MO 68178 USA, and all chemicals were used without further purification.

### 2.2. Preparation of Hydroponically Cultured Ginseng Extracts

The hydroponically cultured ginseng samples were washed with distilled water repeatedly and grounded into fined powder after drying. Five grams of fine powder were mixed with 100 mL of water in a conical flask. The powdered sample was autoclaved at high pressure with maintaining at 100 °C for 40 min to extract the phytochemicals from plant. The autoclaved extracts were filtered with Whatman no.1 filter paper (110 mm) and centrifuged at 4500 rpm for 15 min at room temperature to eliminate undesired components. For the following experiments, the supernatant was stored at 4 °C.

### 2.3. Method of Synthesis GL–CMC–ZnO NCs from Plant Extracts

**Figure 1:**
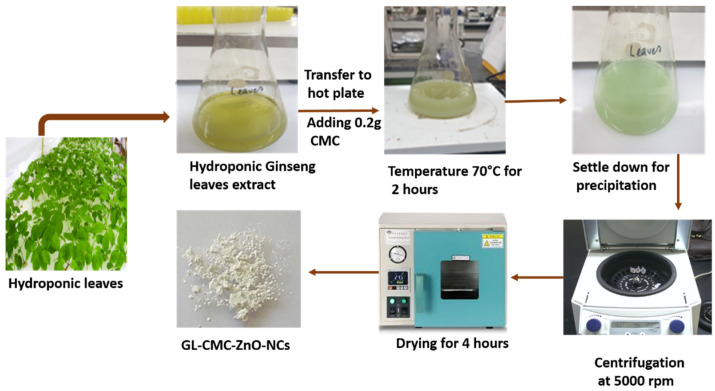
Synthesis method for the preparation of GL–O-carboxymethyl chitosan (CMC)–ZnO nanocomposites (NCs) using hydroponic fresh ginseng leaves with CMC.

To synthesize GL–CMC–ZnO NCs, we followed the co-precipitation method in the presence of chitosan (CMC) with minor modifications described previously [24]. In this method, the hydroponically cultured ginseng plant extract was used as a reducing and coating agent, whereas Zn salt and NaOH were used as precursors. Zinc nitrate salt has higher solubility abilities for developing uniform oxidizing properties for the formation of NPs. Five percentages (*w*/*v*) of the hydroponically cultured ginseng leaf extracts were dissolved with 80 mL of distilled water, and 0.2 g of CMC and 0.1 mM ZnNO_3_ were added to the homogeneous mixture and immediately transferred to a hotplate (70 °C). The solution was stirred using a magnetic stirrer. Then, 0.2 M of a NaOH solution was added dropwise to the homogenous solution to yield white precipitation. After mixing, the reagents solution was stirred continuously at 500 rpm for 2 h. Centrifugation was carried out for 10 min after allowing the mixture to settle for 12 h, and the supernatant was removed. The synthesized NCs were washed with distilled water three times, followed by drying at 60 °C for 4 h to allow Zn(OH)_2_ to be converted to ZnO NPs. The pictorial elaboration is shown in Figure 1.

### 2.4. Characterization of GL–CMC–ZnO NPs

Various analytical methods confirmed the bio-reduction of metal ions to metal NPs. The samples were scanned in the range of 200–800 nm produced by a UV–VIS spectrophotometer (Ultrospec ™2100, 11 Dearborn road, Peabody, MA 01960, USA) and a quartz cuvette (10 mm in length). The FE-TEM analysis was carried out by a tool (JEM-2100 F JEOL, 11 Dearborn road, Peabody, MA 01960, USA) at an operating voltage of 200 kV to check and confirm the morphology of NPs. The essential distribution, purity, and crystallinity of GL–CMC–ZnO NCs were assessed by the selected area diffraction (SAED, JEM-2100 F JEOL, 11 Dearborn road, Peabody, MA 01960, USA), Energy dispersive X-ray spectroscopy (EDX, JEM-2100 F JEOL, 11 Dearborn road, Peabody, MA 01960, USA), and elemental mapping. The GL–CMC–ZnO NCs droplets were put on a carbon-coated copper grid, allowed to dry at 60 °C in an oven and finally transferred to an analyzer. X-ray powder diffraction or XRD analysis was carried out at an operating voltage of 40 kv and a current of 40 mA using the instrument (D8 Advance, Fahrenheitstr., 28359 Bremen, Germany, Bruker, Germany) to check the crystallinity of the NPs. The crystallinity of the GL–CMC–ZnO NCs was confirmed in a 2θ range of 20°–80° with Cu-Kα radiation at a wavelength of 1.54 Å.

The Debye-Scherrer equation was used to check the size of the GL–CMC–ZnO NCs, given as follows:

(1) $$D=\frac{0.9\lambda }{\beta \cos\theta },$$

where, D stands for the size in nm, λ is the wavelength of Cu-Kα in nm, β is the full width at half maximum (FWHM) in radians, and θ is the half of the Bragg angle in radians. The FTIR analysis was scanned in the range of 4000–450 cm^−1^ to analyze the functional group of GL–CMC–ZnO NCs using a Perkin Elmer analyzer (520, South main str. suite, Akron, OH, USA). X-ray photoelectron microscopy (XPS, Thermo Fisher Scientific, Gwangpyeong-ro, Gangnam-gu, Seoul, Korea) was used to check the NCs’ chemical and elemental states and composition by a K-alpha photoelectron spectrometer (Thermo Electron/K-Alpha; Thermo Scientific, Seoul, Korea). The chemical elements state of ZnO NCs was identified by using XPS carried out on a K-alpha (Thermo VG, Kirkton, UK) instrument that was coupled with a monochromatic Al Kα X-ray radiation source with a hemispherical analyzer and measured using an analysis area up to 300 mm × 700 mm. For high-resolution spectra, the electron-energy analyzer was set to a passing energy of 40 eV, and the electron take-off angle (TOA) was set to 90°. The XPS spectra for Zn and O analyses were acquired in a single sweep with an energy step size of 0.1 eV and a dwell time of 300ms^−1^. The high-resolution spectra were background-corrected and fitted using CASA software (version 2.3.19PR1.0; CASA software Ltd., Teignmouth, UK).

The photoluminescence (PL) excitation (PLE) and PL emission properties were investigated using a Sinco FluroMate FS-2 spectrofluorometer (Edinburgh Instruments Ltd., 2 Bain Square, State, Kirkton, UK). The PLE measurements were performed under an emission wavelength of 467 nm. The PL emission measurements were carried out at excitation wavelengths of 229 and 249 nm. The excitation power sources had a luminance of 700 candela.

### 2.5. The Photocatalytic Activity of GL–CMC–ZnO NCs

CR, RB, and MG dyes with a 15 mgL^−1^ concentration were used to check the photocatalytic activity of GL–CMC–ZnO NCs under a UV lamp. The dyes and the catalysts were sonicated for 30 min for proper mixing. Then, the mixture was kept in a dark place to reach the adsorption–desorption equilibrium. The reaction mixture was irradiated by a UV cross-linker (C−1000 Ultraviolet cross-linker with an energy density of 100 µJ/cm) for approximately 10, 20, 25, and 30 min separately. The degradation efficiency was calculated using Equation (2):

Degradation efficiency (%) n = (C_o_ − C_t_)/C_o_, (2)

where, C_t_ is the concentration at time t, and C_o_ is the initial concentration at time t_0_.

## 3. Results and Discussion

### 3.1. UV–VIS Spectroscopy

**Figure 2:**
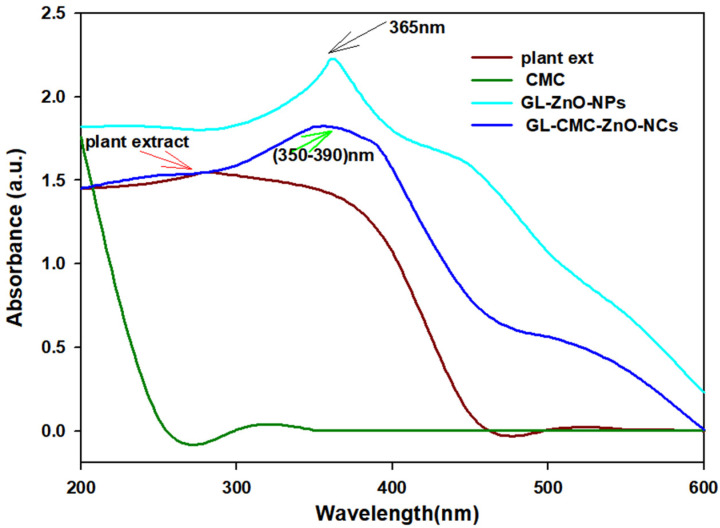
UV–VIS absorbance spectra of hydroponic ginseng leaves ZnO NCs with the CMC polymer. The brown line indicates the result for the plant extracts; the green line indicates the result for the CMC; the violet line indicates the result for the GL–CMC–ZnO NCs; the blue line indicates the result for the GL–ZnO nanoparticles (NPs).

UV–VIS spectroscopy was used to confirm the ZnO NCs from hydroponic ginseng extracts with the CMC polymer. Figure 2 revealed the UV absorbance spectra of hydroponic ginseng zinc oxide NCs with the CMC polymer. The wavelength range was fixed (200–800 nm) to detect the NC formation. The GL–ZnO NPs showed a sharp surface resonance peak at ~365 nm to confirm the GL–ZnO NPs formation, and the leaves extract alone showed a peak near 250–280 nm to confirm the presence of the polyphenolic compound. The GL–CMC–ZnO NCs showed a broader peak at 350–380 nm.

The broader distribution may form due to the less hydrophilic plant extract absorption and the tightly bounded amine group with metal ions [31].

### 3.2. FE-TEM Analysis

**Figure 3:**
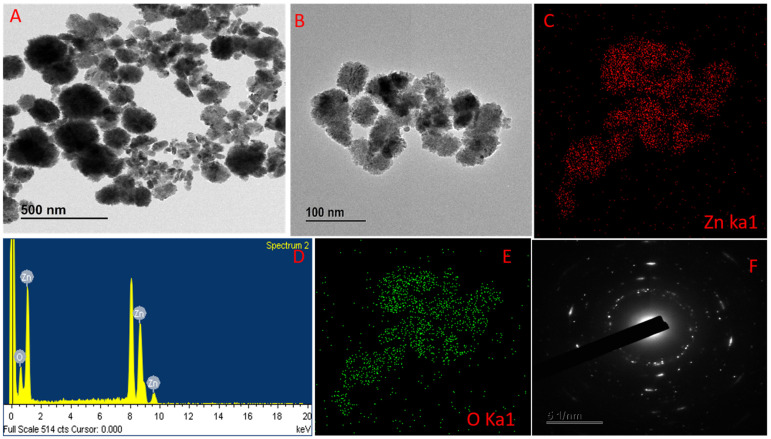
(**A**,**B**) FE-TEM images of GL–CMC–ZnO NCs. (**C**) Zinc elemental distribution of GL–CMC–ZnO NCs. (**D**) Energy dispersive X-ray spectroscopy (EDX) spectra of GL–CMC–ZnO NCs. (**E**) Oxygen elemental distribution of GL–CMC–ZnO NCs. (**F**) SAED structure of GL–CMC–ZnO NCs.

The NCs formation image of the synthesized GL–CMC–ZnO NCs using FE – TEM analysis is shown in Figure 3A, B. The nanoformulation was oval-cotton-shaped. Figure 3A,B shows the good dispersion of the NCs due to the capping effect of the extracts and the CMC, which prevented the cluster of the formulation. The high crystallinity of the GL–CMC–ZnO NCs was revealed by an SAED pattern (Figure 3F). The elemental mapping showed the distributions of zinc (red dot) and oxygen (green dot) in Figure 3C,E and Table 1. The weight percentage ratios of both oxygen and zinc was 18.26% and 81.74%, respectively. The atomic percentages of zinc and oxygen were recorded at 52.29% and 47.71%, respectively. EDX spectroscopy was used to determine the purity of GL–CMC–ZnO NCs, as shown in Figure 3D. The EDX spectra identified Zn and O that demonstrated the formation of ZnO NCs. No extra peaks were detected for other elements. The SAED pattern showed the hexagonal ring of spots, demonstrating the crystallite nature of the GL–CMC–ZnO NCs.

### 3.3. XRD Analysis

**Figure 4:**
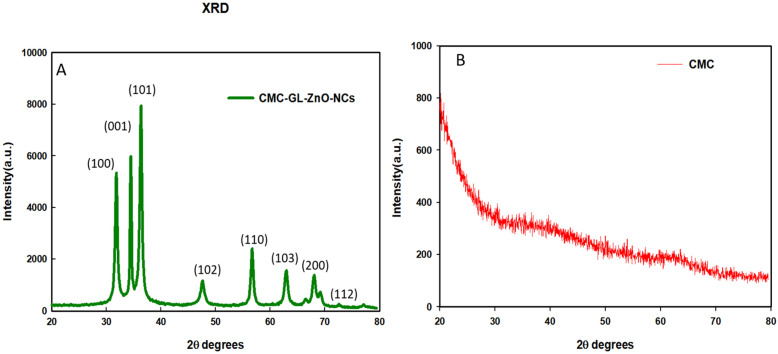
XRD patterns of the GL–CMC–ZnO NCs (**A**) and the polymer CMC (**B**).

The XRD pattern demonstrated the crystallite size of the NCs by calculating the FWHMs of the three most intense diffraction peaks. The cotton-shape GL–CMC–ZnO NCs were composed of ZnO NPs with an average crystallite size of 28 nm (Table 2). The position, width, peak intensity, and FWHM data were calculated at a 2θ range of 20°–80°, which were indexed according to Miller indices (*h*, *l*, *k*) at (100), (001), and (101) lattice planes, respectively [32]. Figure 4A,B shows the XRD patterns of the GL–CMC–ZnO NCs and the CMC, respectively, where the CMC exhibited a broad diffraction peak at a 2θ range of 20°–35°, which confirmed the amorphous nature of the polymer. The hexagonal crystallite structure is thoroughly agreeable with the standard value JCPDS NO. 36−145 of ZnO nanocrystal [33]. In Figure 4A, the XRD pattern of the GL-CMC-ZnO NCs showed a hexagonal wurtzite structure of bulk ZnO with no extra peak that confirmed the purity of the ZnO NCs [34].

### 3.4. FTIR Analysis

**Figure 5:**
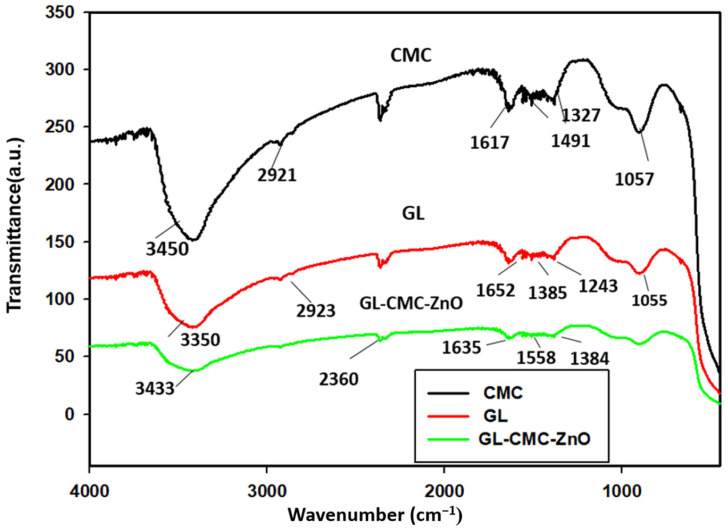
FTIR spectra of GL–CMC–ZnO NCs (green line), CMC (black line), and ginseng leaves extracts (red line).

The phytochemicals responsible for the NP stability were diagnosed using FTIR spectroscopic analysis in Figure 5. The FTIR spectra revealed the phytochemicals present in the GL–CMC–ZnO NCs, which was responsible for forming stable GL–CMC–ZnO NCs from the extracts. The FTIR spectra showed the characteristics peaks of the GL–CMC–ZnO NCs, hydroponic ginseng leaf extracts, and the CMC polymer alone. The GL–CMC–ZnO NCs exhibited a 3433 cm^−1^ broad peak corresponding to hydroxyl –OH and N–H due to the attached polyphenol of ginseng and the secondary amine of the CMC polymer. The 2900 cm^−1^ bond of NCs is due to the aliphatic (C–H) chain of the CMC polymer and the plant extracts. The absorbance peak at 1635 cm^−1^ is due to the carbonyl (C=O) and secondary amide (N–H) groups from the polymer and the plant extracts. The absorbance peak at 1600–1300 cm^−1^ corresponding to O–H and C–H bonds and also 1055–1350 cm^−1^ for the C–H aliphatic ether group for the polymer CMC [32]. The absorption peak at 450 cm^−1^ confirmed a Zn–O metal bond that confirmed the formation of GL–CMC–ZnO NCs from ginseng leaves and the CMC polymer. However, there was no peak observed in the zone of 400–500 cm^−1^ in GL extracts [35].

### 3.5. XPS Analysis

**Figure 6:**
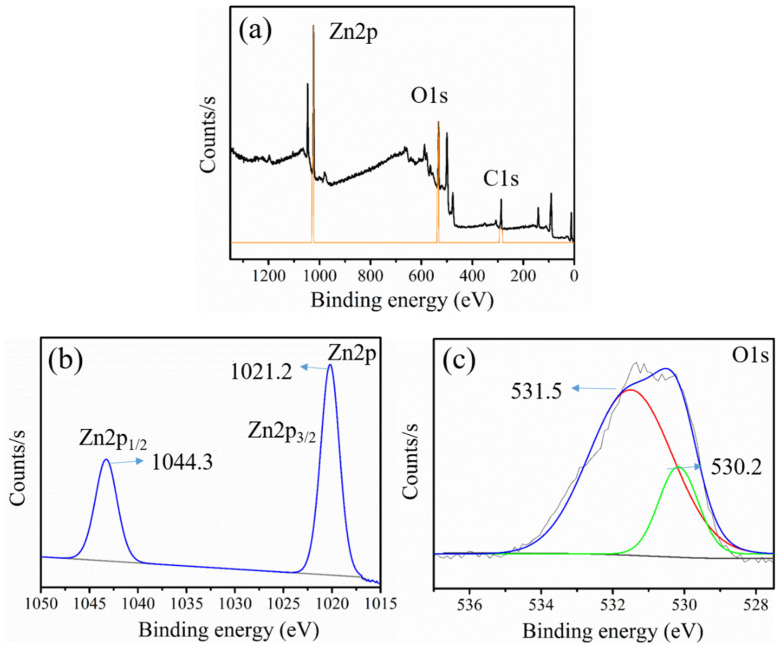
X-ray photoelectron spectroscopy (XPS) analysis of the GL–CMC–ZnO NCs: (**a**) full survey spectrum; (**b**) Zn2p binding energy region; (**c**) O1s binding area.

The chemical state of elements in ZnO was analyzed using XPS analysis, as shown in Figure 6. The full survey spectra displayed in Figure 6a suggested the presence of three major elements, i.e., Zn, O, and C, in the GL–CMC–ZnO NCs. The C1s peak in Figure 6a indicated the presence of C traces, which perhaps appeared to be from the plant extracts. The binding energy peaks appearing at 1021.2 and 1044.3 eV in Figure 6b were attributed to Zn2p3/2 and Zn2p½, respectively, suggested the existence of the Zn^2+^ state [36]. Besides, Figure 6c shows the binding energies of O1s at 530.2 and 531.5 eV were ascribed to Zn–O and C=O sites, respectively.

### 3.6. PL

**Figure 7:**
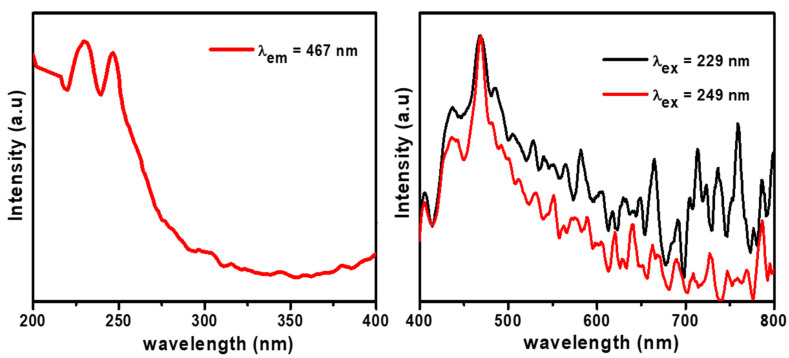
Photoluminescence analysis results of the GL–CMC–ZnO NCs.

PL mainly indicates the essential parameters of semiconductors, primarily structural defects, energies defects, and impurities. The PL excitation band of GL–CMC–ZnO NCs was measured at room temperature. The PL excitation measurements were performed under an emission wavelength of 467 nm. The PL emission measurements were carried out at excitation wavelengths of 229 and 249 nm, as shown in the Figure 7. The PL study of the NCs indicated the intense peaks at 405, 445, 480, and 549 nm due to the deep level effect. The intense peak at 480 nm is due to the electronic transition from the interstitial level of zinc to the valence band [33]. The intense peak at 480 nm (blue broad emission peak) is responsible for recombining an electron with oxygen vacancy [37]. Besides, the green emission at 549 nm is the recombination of an electron with an oxygen vacancy (V_0_) and photo-generated holes. The PL study of GL–CMC–ZnO NCs showed the slower the electron–hole recombination that accelerated the dye molecule degradation.

### 3.7. Catalyst Loading Efficiency

**Figure 8:**
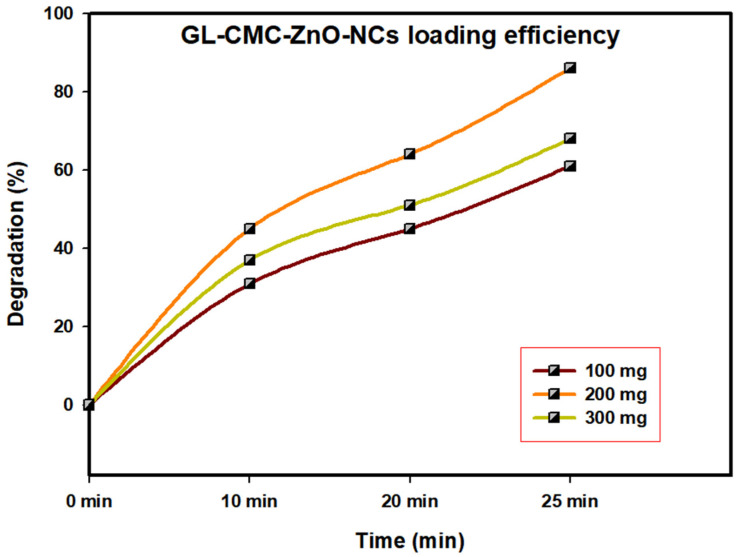
GL–CMC–ZnO NCs nanocatalyst loading optimization using different concentrations.

The GL–CMC–ZnO NCs act as a catalyst in different dye degradation rates under UV illumination; here, MB dye was checked for degradation by catalyst under UV illumination during the optimization process (Figure 8). At a concentration of 100 mg/L, the catalyst concentration was checked in different time intervals (0 min, 10, 20, and 25 min). Further, the concentration of the catalyst was increased from 100 to 300 mg/L to check the degradation rate. When the 300 mg/L catalyst was checked, the gradual degradation rate was lower than for the 200 mg and 100 mg mg/L catalysts. It may be due to the overlapping tendency of the catalyst upon dye molecules that restrict their interaction. The complete optimization process confirmed that the 200 mg/L catalyst concentration showed the highest degradation rate of dye molecules.

### 3.8. The Photocatalytic Activity of the GL–CMC-ZnO NCs

The GL–CMC–ZnO NCs were used as a photocatalyst to degrade the three types of industrial dyes (MG, CR, and RB) at a 15 mg/L dye concentration under UV illumination. The UV cross-linker (C-1000 ultraviolet cross-linker, energy: 100 µJ/cm^2^ (364 nm)) was used as an illuminator in different time intervals. The MG, CR, and RB degraded by 96%, 87%, and 94% within a 30 min contact time, respectively.

**Figure 9:**
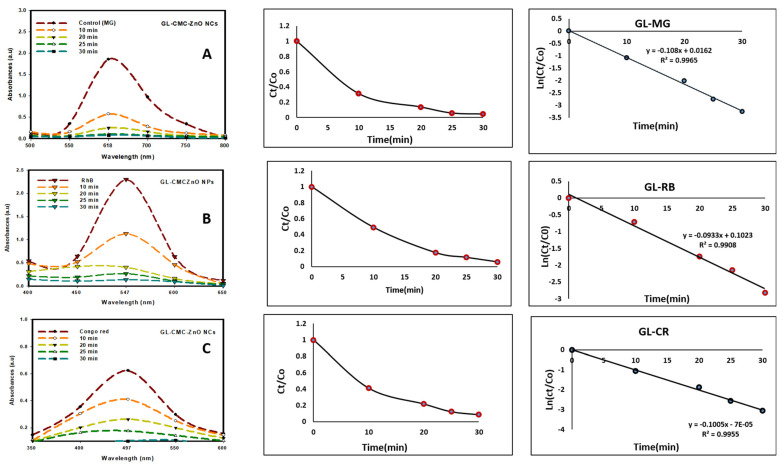
Photocatalytic activities of GL–CMC–ZnO NCs under UV irradiation with the kinetic study: (**A**) malachite green (MG) degradation with kinetic study; (**B**) Rhodamine B (RB) dye degradation with kinetic study; (**C**) Congo red (CR) dye degradation with kinetic study.

**Figure 10:**
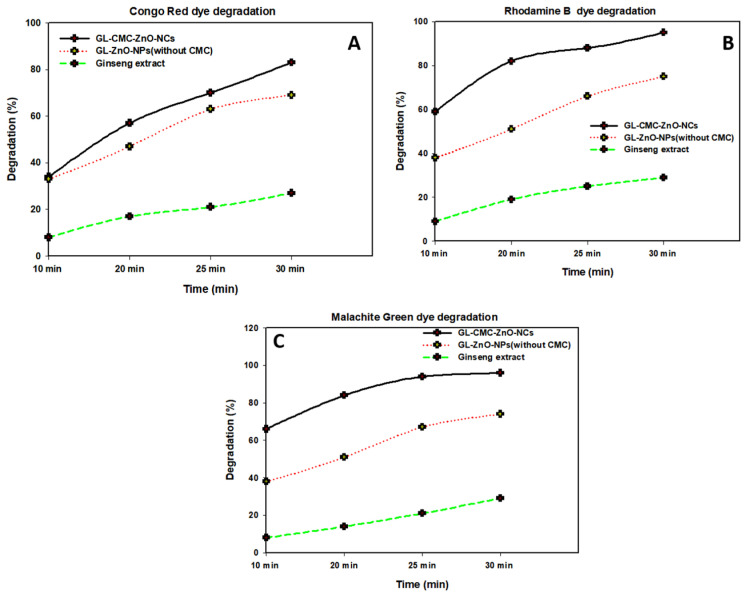
Different dye degradation rates with time using the GL–CMC–ZnO NCs: (**A**) CR dye degradation rates; (**B**) RB dye degradation rates; (**C**) MB dye degradation rates.

**Figure 11:**
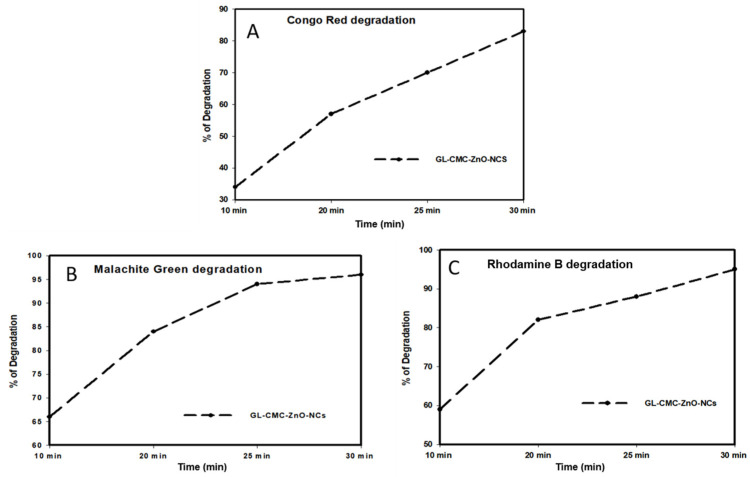
Comparison study for the dye degradation rates using the GL–CMC–ZnO NCs: (**A**) CR degradation rates; (**B**) MB degradation rates; (**C**) RB dye degradation rates.

Figure 9 shows the kinetics study of three dyes following the 1st-order kinetics model confirmed from linear regression (>0.99), and the degradation of dyes using NCs was compared with leaves extracts and ZnO NPs (without CMC). The results suggested that the GL alone degraded by approximately 30% and ZnO NPs degraded by approximately 71% in the case of three dyes in Figure 10 and the dye degradation rates was given in Figure 11. The degradation rate increased due to the combination of the polymer CMC with ZnO NPs to form NCs that interacted with dye molecules more by creating a more active site. The hydroponic ginseng leaves contained polyphenol and ginsenoside, and the CMC polymer had the ability to dissolve ginsenoside [24], which mainly increased the catalyst efficiency. Besides, the CMC polymer enhanced the NC’s absorption capability, increasing the catalytic efficiency [38]. Besides, the blank experiment was performed with UV light (supplementary data Figure S1). The mechanism of dye degradation can explain that the GL–CMC–ZnO NCs acted as semiconductors, as evidenced by the PL study. The electron–hole generation process is mostly responsible for the dye degradation process. The UV light then passed through the NCs, creating an electron and holes due to photoexcitation. The holes reacted with water molecules form hydroxyl radicals. The possible mechanism is shown as:

GL–CMC–ZnO NCs + hv → e^−^ + h^+^ + Nanocomposite.

The oxidation reaction upon the nanocatalyst is shown as:

h^+^ (VB) + H_2_O → OH + H^+^,

2h^+^ + 2H_2_O → 2H^+^ + 2H_2_O_2_.

Further, the electron reacted with the oxygen molecules to form superoxide’s and hydroxyl radicals reacted with dye molecules to degrade toxic dyes, whereas CO_2_ and H_2_O form as the by-product, as shown below:

e^−^ (CB) + O_2_ → O2^•−^,

Dye molecules (MG, CR, and RB) + (O2^•–^·OH) → CO_2_ + H_2_O + degraded product.

### 3.9. Catalyst (GL–CMC–ZnO NCs) Reusability

**Figure 12:**
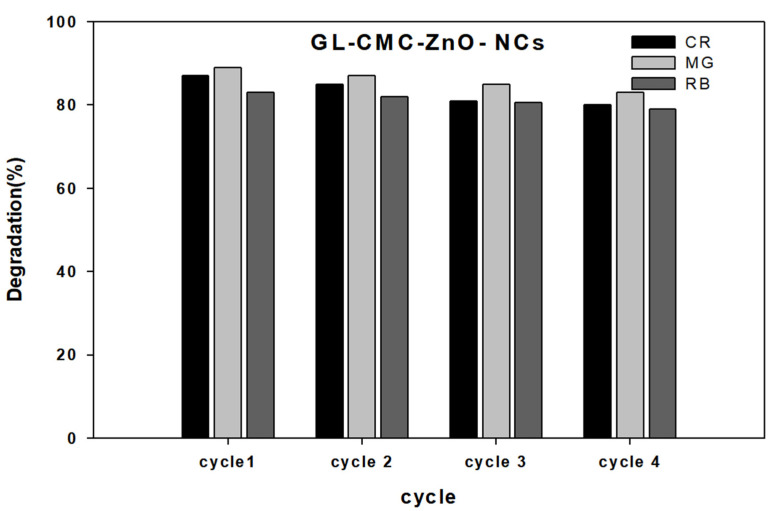
Reusability test results for the GL–CMC–ZnO NCs using dyes (CR, MG, and RB).

Catalyst reusability is a significant parameter for degrading dye molecules or any toxic substances in water purification. Here, the NC was checked with reusability tests by centrifugation, washing, and drying in an oven for further use in Figure 12. The NCs were used four times. The test results highlighted that the NCs could act as a catalyst more than four times without losing significant dye degradation ability (slight decline due to washing). Further, the NCs were characterized by FE-TEM to see the structures, but there were no structural changes happening after four cycles of washing (supplementary data Figure S2).

## 4. Conclusions

This study revealed the synthesis of an eco-friendly NC using hydroponic ginseng leaves and the natural polymer CMC to degrade different dyes (MG, CR, and RhB with different time intervals. The ginseng leaves o-carboxyl methyl chitosan zinc oxide NC (GL–CMC–ZnO NCs) acts as a semiconductor that creates electrons and holes due to photoexcitation under UV Illumination evidenced with the PL study. The slower the electron–hole regeneration process, the faster the dye degradation. After the synthesis, the NC was characterized with different analytical methods. The XPS analysis revealed the chemical composition (C, Zn, and O) presence in the catalyst. The UV analysis and FTIR spectra confirmed the presence of the CMC polymer and polyphenol from the ginseng extracts.

The further EDX analysis evidenced the purity of the nanocatalyst. The FE-TEM images showed cotton-shaped NCs, and the XRD analysis demonstrated the crystallite size of the NC of about 28 nm. The GL–CMC–ZnO NCs at 15 mg/L were used to degrade MG, CR, and RB dyes by 95%, 87%, and 94%, respectively, at a total 30 min time interval. This research highly recommends the eco-friendly GL–CMC–ZnO NCs as an efficient nanocatalyst in large-scale wastewater treatment.

## Figures and Tables

**Table 1:** The percentages of zinc and oxygen in the GL–CMC–ZnO NCs through EDX analysis.

Element	Weight (%)	Atomic %
Zn K	81.74	52.29
O K	18.26	47.71
Total	100.00	100.00

**Table 2:** The crystallite sizes of GL–CMC–ZnO NCs using XRD analysis.

Number of Peak	Peak Position (2θ)	Full Width at Half Maximum (FWHM)	Size (nm)	Average Size (nm)
100	31.72	0.2255	27.10	
002	34.52	0.2241	29.59	28.41
101	35.27	0.2155	28.54	
102	47.45	0.4314	17.26	
110	56.49	0.3497	24.97	
103	62.67	0.451	18.95	
112	68.92	0.5782	16.10	

## Data Availability

This data is available only request on corrosponding author.

## Supplementary Materials

The following are available online https://www.mdpi.com/article/10.3390/ma14216557/s1. Figure S1. The dye degradation rate with and without UV light (A) Rhodamine B (RB) dye; (B) Congo Red dye (CR) & (C) Malachite Green Dye (MG). Figure S2. The FE-TEM images of GL-CMC-ZnO-NCs using a scale bar (A) Size 111.4 nm; (B) Size 91.23 nm.
