# Supplementary material for: Hydroponic Cultured Ginseng Leaves Zinc Oxides Nanocomposite Stabilized with CMC Polymer for Degradation of Hazardous Dyes in Wastewater Treatment
Source: Materials (Basel). 2021 Nov 1;14(21):6557. doi: 10.3390/ma14216557 (PMC8585460; doi:10.3390/ma14216557)
Supplement: Supplementary file 1 [file materials-14-06557-s001.zip › materials-1394190-supplementary.pdf]

Supplementary data

Manuscript: Materials_ 1394190

Title: Hydroponic Cultured Ginseng Leaves Zinc Oxides Nanocomposite Stabilized with CMC Polymer for Degradation of Hazardous Dyes in Wastewater Treatment

Author: Yinping Jin [1,†], Ling Li [2,†], Reshmi Akter [2], Esrat Jahan Rupa [2], Deok-Chun Yang [2], Se Chan Kang [2,*] and Hao Zhang [1,*]

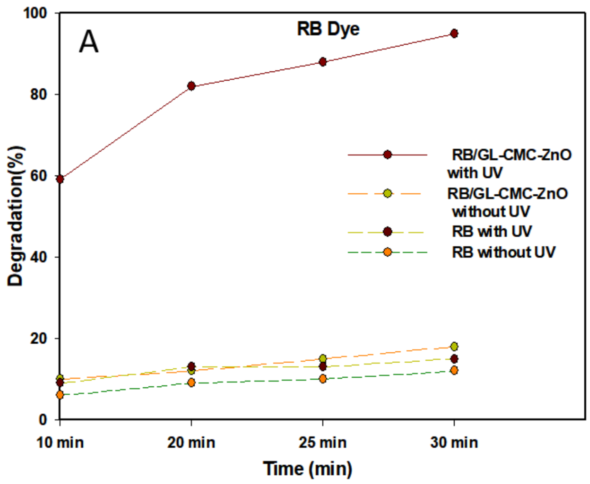

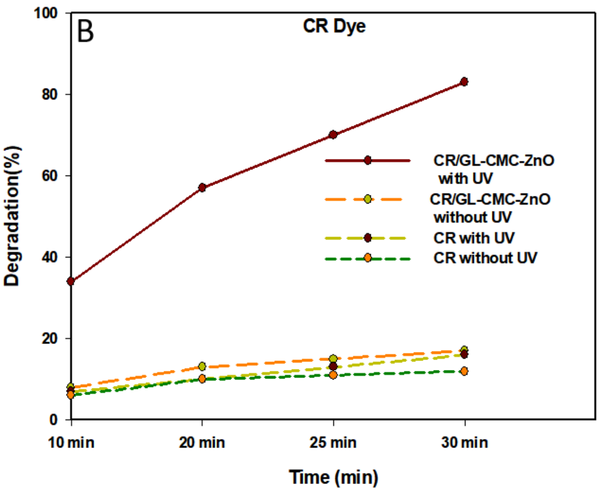

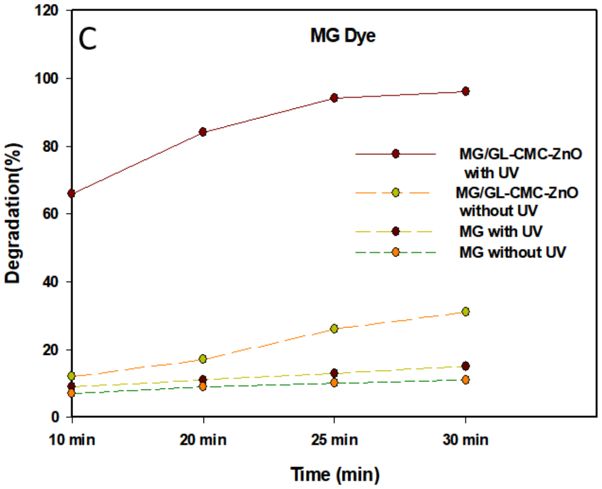

Figure S1.The dye degradation rate with and without UV light (A) Rhodamine B (RB) dye; (B) Congo Red dye (CR) & (C) Malachite Green Dye(MG)

Here, the photocatalytic performance of GL-CMC-ZnO-NCs was investigating under UV light and without UV light. The Nanocomposites show the highest dye degradation rate under UV light. The photocatalyst

degraded Congo Red(CR), Rhodamine B(RB), and malachite Green (MG)dyes approximately 87%, 94%, and 96% within 10, 20, 25, and 30 min of contact time, respectively. But in the absence of UV light, the degradation rate is shallow. Besides, without catalyst, the dye degradation rate is very poor.

FE-TEM images using scale bar:

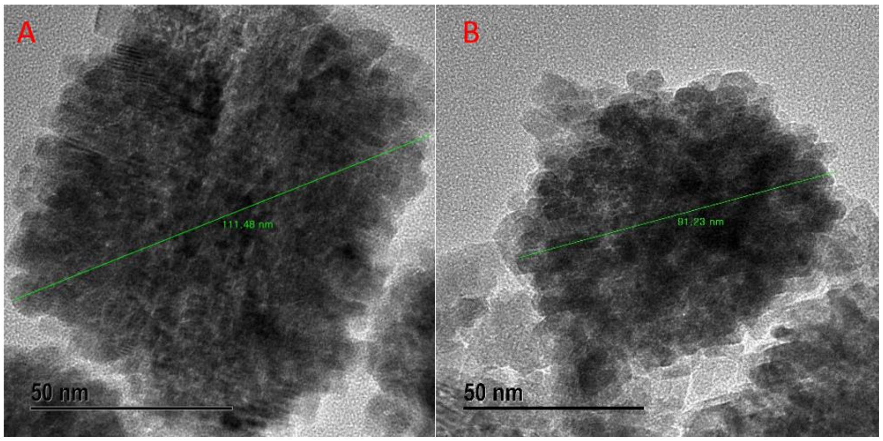

Figure S2. The FE-TEM images of GL-CMC-ZnO-NCs using a scale bar (A) Size 111.4nm; (B) Size 91.23nm.

Here, measuring the size using scale ber shows the size is around (90-112)nm. XRD shows the crystalline core size of the nano composite so the size is small than FE-TEM.
